# A Comprehensive Operation Status Evaluation Method for Mining XLPE Cables

**DOI:** 10.3390/s22197174

**Published:** 2022-09-21

**Authors:** Yanwen Wang, Peng Chen, Yanying Sun, Chen Feng

**Affiliations:** 1 School of Mechanical, Electronic & Information Engineering, China University of Mining and Technology-Beijing, Beijing 100083, China; 2 The Key Laboratory of Power System Intelligent Dispatch and Control, Shandong University, Jinan 250061, China

**Keywords:** status evaluation, mining XLPE cables, membership cloud, D-S evidence theory

## Abstract

At present, the online insulation monitoring and fault diagnosis of mining cables are extensively discussed, while their operation status assessment has not been deeply studied. Considering that mining cables are closely related to the safe and stable operation of coal mine power supply systems, a comprehensive evaluation method including the Analytic Hierarchy Process (AHP), the membership cloud theory, and the D-S evidence theory is proposed in this paper in order to accurately assess the operation status of the mining XLPE cable. Firstly, the membership cloud is introduced to solve the index membership degree and the weights are calculated by an improved weight vector calculation method. Secondly, the conversion from the base layer indicator membership degree to the target layer trust degree is realized based on the D-S evidence theory. Then, the cable operation status is judged via the trust degree maximum and the distribution of conflict coefficients is further analyzed to warn the indicators with a bad status in the base layer. Finally, the feasibility of the proposed evaluation method is verified by a sufficient and detailed case analysis.

## 1. Introduction

As an important component of a coal mine’s power grid, the mining XLPE cable (hereafter referred to as mining cable) is the core component of a coal mine’s power supply system, and its operational safety status directly affects the stable operation of the coal mine’s power grid and even concerns the production safety of the coal mine itself. The operating safety status of these mining cables is mainly affected by their operating environment and operating conditions [1]. Unlike the operating environment of cables in ordinary power grids, the air humidity in underground coal mines is high and the temperature varies greatly in different areas. Because of this, the insulation in mining cables is easily aged which leads to insulation degradation, and the space in underground coal mines is narrow, making mining cables susceptible to smashing, touching, and dragging, which cause the cable insulation to be damaged, wherein grounding or leakage faults can occur. The grounding method of the neutral point through to the arc extinguishing coil is usually used in a coal mine’s power grid, although this grounding method can, in principle, allow the power supply system to operate with faults for 2–3 h. However, the underground environment of coal mines is different from that on the ground. The closed underground environment is filled with a large amount of gas and coal dust. When the mine cable discharges due to insulation failure or single-phase grounding, the generated sparks can easily lead to the environment catching “fire” and/or ”explosive” conditions which could cause serious coal mine safety accidents such as electromechanical accidents, cable “release”, underground fires, and explosions [2,3,4,5]. According to the national and provincial coal mine accident analysis reports released in 2021, there were 122 coal mine accidents and 225 fatalities in 2020 of which electromechanical, gas, and cable discharge accidents accounted for 19% of the total number of accidents and 27% of the total number of fatalities, with some provinces accounting for a higher percentage of electromechanical, gas, and cable discharge accidents than the national accident rate. In some provinces, the proportion of electromechanical, gas, and cable discharge accidents was higher than that of average national accidents, and, among these, cable failure in coal mines was a direct cause of cable discharge accidents, an important factor that led to a number of electromechanical accidents and was a major external source of ignition-causing gas accidents.

Mining cable faults pose a long-term threat to the economic, safe, and reliable operation of coal mine power grids. Therefore, it is crucial to diagnose the operation status and fault monitoring of mine cables within coal mines. If we can intelligently sense and evaluate the operational safety status of cables in a coal mine power grid in real-time and accurately detect abnormalities and warnings of faults before they occur in order to prevent accidents before they happen, this will break through the current technical bottleneck and, with a small amount of investment, solve the actual demand problem and effectively reduce the personal and property losses caused by cable faults. This is a necessary precondition to ensure the safety of coal mine production and can bring about strong economic and social benefits and broad application prospects with significant research necessity and urgency.

In order to achieve an accurate assessment of the operating status of mining cables, this paper establishes a mining cable evaluation index system using hierarchical analysis based on a combination of expert industry opinions, relevant literature, protocols, and research. We first divide the mining cable status into severe, abnormal, attention, and normal, and then transform the standard status level into a visualized status space according to the cloud model theory; then, we calculate the membership degrees of quantitative and qualitative indicators, respectively; then, we use the improved AHP weight calculation algorithm to calculate the weights of each indicator in the indicator layer; and, finally, in order to reduce the uncertainty within the evaluation process, the fusion of the indicator membership and weights is realized step-by-step based on the D-S evidence theory and the current cable status is judged based on the fusion results.

The structure of this paper is as follows: Section 2 provides a literature review and summarizes previous research results; Section 3 introduces the AHP algorithm, the improved AHP weight calculation algorithm, and the D-S evidence theory; Section 4 describes the mining cable condition evaluation system and the calculation of the membership degrees of quantitative and qualitative indicators; Section 5 presents the detailed numerical work in the evaluation model; and, finally, the conclusions of this paper are given in Section 6.

## 2. Literature Review

Currently, cross-linked polyethylene (XLPE) power cables are widely used because of their excellent insulation and heat resistance properties [6,7,8]. Due to uncertainties in the design and production process, the frequency of faults has gradually increased, reducing the safety of the power grid. Cable insulation or fault monitoring methods mainly include temperature, DC components, dielectric loss, partial discharge, and traveling wave detection methods [9,10,11]. DC component and dielectric loss methods can only be used to perform overall insulation condition assessment with low accuracy. The local discharge method is influenced by the surrounding environment and signals propagation distance [12]. High-frequency signals decay rapidly making long-distance measurements difficult. Temperature monitoring methods are not sensitive enough for fault identification and the data generated by them is of limited use. For the traveling wave method, it is difficult to detect cable faults because the amplitude of the wave head is significantly attenuated after the reflected wave propagates through the long cable, and it is easily affected by the interference signal. To make up for the shortcomings of the previous methods, many novel methods for online cable monitoring have been proposed by many experts and scholars in recent years. Guangya Zhu [13] proposed a new online monitoring method of power cable insulation conditions based on low-frequency signal injection. For this method, a low-frequency signal is injected into the power system via the potential transformer’s (PT) open delta configuration. The cable conductor voltage and leakage current are detected. The interpolating windowed fast Fourier transform (FFT) algorithm is applied to calculate the dielectric loss angle. Then, the cable tangent delta (tanδ) can be deduced and the cable condition can be assessed. Wei Zhao [14] drew a two-dimensional trajectory map by simultaneously measuring two circulating currents in a coaxial cable. Fault criterion and databases are established to detect faults by analyzing the changes in trajectory characteristic parameters. Wenxia Pan [15] proposes a distributed online monitoring method for cable PD based on the phase-sensitive fiber-optic time domain reflection (φ-OTDR) principle. When the cable has PD, the backscattered Rayleigh light intensity change of the PD position is higher than the intact position. Yang Wu [16] proposed a monitoring scheme based on CM leakage current measurements at selected monitoring frequencies and developed an aging feature extraction method based on principal component analysis (PCA) which provides an estimate of the insulation’s aging severity.

However, the online monitoring of the cable is only for online monitoring of a certain index of the cable and does not evaluate the overall running status of the cable in many aspects. In response to such problems, more and more scholars put their research focus on cable condition assessment. Heqian Liu [17] introduced the theory of dielectric response such as the isothermal relaxation current. Combined with the cable-aging equivalent model, the isothermal relaxation current peak-split fitting method, to represent the different processes of relaxation according to the attenuation characteristics of isothermal relaxation current, is provided. Lulu Li [18] proposed a non-invasive aging assessment method using transient disturbances originating from the system. The relative dielectric constant of the cable is extracted from the response of transient disturbances instead of the conventional dielectric loss angle in order to characterize the aging state more sensitively. Yanqun Liao [19] proposed a novel holistic approach in order to facilitate the implementation of risk-based maintenance strategies for cable conduits, cable terminations, joints, bodies, and grounding systems for each cable loop. Based on the polymer trap theory and the extended Debye model, the shape of the PDC curve, depolarization charge, parameters of the extended Debye model, aging factor (*A*), elongation at break retention rate (EB%), and their relationships under different thermal aging degrees were analyzed by Yiyi Zhang [20]. It is worth noting that there are many factors that affect the running state of the cable and no relevant scholars have carried out an in-depth analysis of the factors affecting the running state of the cable in all aspects and angles. Therefore, an index system that can scientifically evaluate the running state of the cable has not yet been established. At the same time, the comprehensive evaluation method of cable operation status also needs to be studied more deeply.

## 3. Evaluation Models and Principles

### 3.1. AHP Method

AHP is a multi-criteria decision-making method (MCDM) developed by T.L. Satty in order to evaluate and select alternatives based on a set of selected criteria [21]. This process can combine judgments from intangible qualitative criteria with tangible quantitative criteria. The specific steps of the AHP are as follows:

**STEP 1:** Establish a hierarchical structure.

We need to stratify the problem to be analyzed and establish a three-level structure model including the target layer, factor layer, and base layer.

**STEP 2:** Construct a comparison judgment matrix.

(1) $$A=\left[\begin{array}{cccc} a_{11} & a_{12} & \cdots & a_{1n}\\ a_{21} & a_{22} & \cdots & a_{2n}\\ \vdots & \vdots & \ddots & \vdots \\ a_{n1} & a_{n2} & \cdots & a_{nn} \end{array}\right]$$

We need to construct a matrix ***A*** according to the relative importance of each indicator. The basis for judging relative importance is shown in Table 1. *a_ij_* represents the importance of the *i*-th indicator relative to the *j*-th indicator. When *i* = *j*, there is *a_ij_* = 1; otherwise, there are *a_ij_* > 0 and *a_ji_* = 1/*a_ij_*.

**STEP 3:** Calculate the weight vector.

(2) $$\omega_{i}=\frac{{\left(\prod_{j=1}^{n}a_{ij}\right)}^{\frac{1}{n}}}{\sum_{k=1}^{n}{\left(\prod_{j=1}^{n}a_{kj}\right)}^{\frac{1}{n}}}$$

According to Formula (2), ***W*** = [*ω*_1_, *ω*_2_, …, *ω_n_*]^T^ can be calculated.

**STEP 4:** Consistency test.

In order to verify whether the weight vector is reasonable, we need to check its consistency. Formulas for calculating the random consistency ratio *CR* are as follows:

(3) $$CR=\frac{CI}{RI}$$

(4) $$CI=\frac{\lambda_{\max}-n}{n-1}$$

(5) $$\lambda_{\max}=\frac{1}{n}\sum_{i=1}^{n}\frac{{\left(AW\right)}_{i}}{\omega_{i}}$$

where (***AW***)*_i_* is the *i*-th element of the product of ***A*** and ***W***.

The average random consistency index RI of the multilevel matrix can be obtained from Table 2.

If *CR* < 0.1, the comparison judgment matrix has a satisfactory consistency index; otherwise, the comparison judgment matrix needs to be readjusted.

In order to solve the problem that AHP is too subjective, we propose an improved weight vector calculation method. The method replaces the comparison judgment matrix with the interval judgment matrix and searches for the matrix with the highest consistency ratio in the interval to calculate the weight vector. This can reduce the subjectivity of experts and improve the objectivity of weights. The specific weight vector calculation has been introduced in detail in our previous study, and, therefore, in this paper, we will not describe too much due to the limitation of space and the details can be referred to in the literature [22].

### 3.2. Membership Cloud Theory

The membership cloud is a model proposed by academician Deyi Li in 1995 for converting qualitative evaluation and quantitative values, which represents the conversion relationship between numerical and linguistic values and can take into account the randomness and fuzziness of linguistic evaluation. The fuzziness is described by the width of the cloud and the randomness is described by the thickness of the cloud [23].

The definition of the membership cloud is: let *U* be an exact numerical representation of the universe of discourse. In the corresponding qualitative concept *A* on *U*, for any element x in the universe, there is a random number *y* ∈ [0, 1] with a tendency to be stable, which is called the membership degree of *x* to *A*, and the distribution of the membership degree in the universe is called the membership cloud, referred to as a cloud. Clouds are composed of several cloud droplets. Cloud droplets are quantitative descriptions of qualitative concepts. The generation process of cloud droplets expresses the uncertainty mapping relationship between qualitative concepts and quantitative values. According to the dimension of the universe *U*, a cloud can be divided into a one-dimensional cloud, a two-dimensional cloud, a multi-dimensional cloud, and so on.

The portrayal of the cloud model relies on three parameters, which are expectation *Ex*, entropy *En*, and super entropy *He*. Among these, *Ex* reflects the central value of a concept corresponding to a theoretical domain, *En* reflects the ambiguity of the concept, and *He* reflects the discrete degree of the cloud drops. The forward cloud generator forms a number of random numbers with stable tendencies based on the numerical characteristics of the cloud model to form an evaluation cloud; the inverse cloud generator calculates the numerical characteristics of the cloud model based on finite expert evaluation.

### 3.3. D-S Evidence Theory

In the 1960s, Dempster proposed the concept of evidence theory and his student Shafer redefined it and created the “mathematical theory of evidence”, which was later called D-S evidence theory. Because D-S evidence theory has the ability of uncertainty reasoning and can represent, fuse, and decide uncertain information, it has been widely used in the fields of decision analysis, pattern recognition, and information fusion. The basic principles of D-S evidence theory are as follows [24,25]:

Assuming that *U* is the identification frame, it is a finite and complete universe of discourse, and *A* is a subset of *U*. If there is a set function m:P(*U*)→[0, 1] that satisfies the condition of Formula (6):

(6) $$\{\begin{array}{c} \sum_{A\subseteq U}m(A)=1\\ m(\emptyset )=0 \end{array}$$

where *m* is the probability distribution function on the identification frame *U*. When *m*(*A*) > 0, *A* is a focal element; *m*(*A*) is the function value of the probability distribution function corresponding to event *A*. When the identification frame *U* is incomplete, *m*(∅) ≠ 0. This paper only discusses the case when the identification frame *U* is complete and the elements are limited.

Dempster’s rule for fusing N pieces of evidence is shown in Formula (7):

(7) $$m(A)=\frac{1}{1-K}\sum_{A_{1}\cap \ldots A_{M}=A}\prod_{i=1}^{N}m_{i}(A_{i})$$

In Formula (7):

(8) $$K=\sum_{A_{1}\cap \ldots A_{M}=\emptyset }\prod_{i=1}^{N}m_{i}(A_{i})$$

where *K* represents the conflict size between the evidence bodies. When *K* = 1, the combination rule is invalid and the evidence bodies completely conflict. When *K*→1, the fusion decision result may be contrary to common sense, so effectively resolving evidence conflicts is an important part of obtaining reliable fusion results.

## 4. Empirical Application of the Evaluation Model

In accordance with the requirements of AHP, we analyzed the main factors affecting the operation status of cables for coal mines, determined the interval judgment matrix of each layer, and obtained the optimal weight vector through the improved AHP algorithm. The membership matrix of each layer to the target layer was established through the membership cloud theory and the optimal weight vector was synthesized with the membership matrix by evidence synthesis through the D-S evidence theory in order to obtain a comprehensive judgment on the operating state of the mine cable.

### 4.1. Section of the Voltage Situation Evaluation

**Figure 1:**
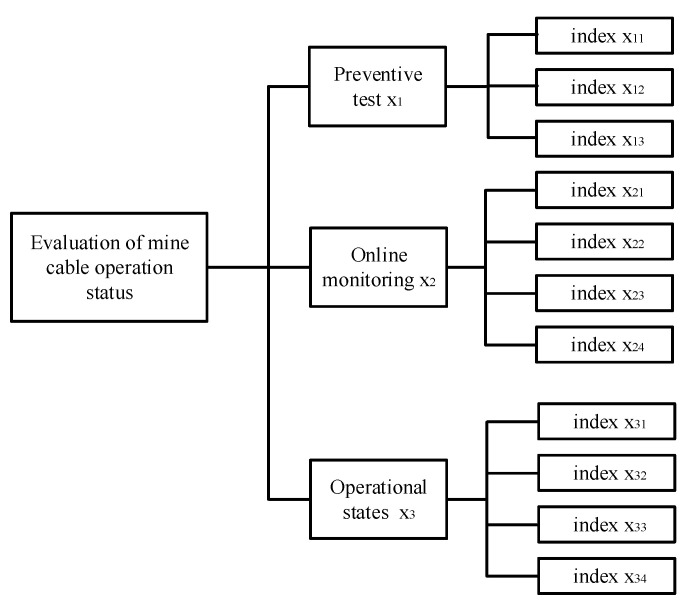
Operation status evaluation model for mining XLPE cables.

The operating condition of mining cables is influenced by a variety of factors. The selection of evaluation indexes plays a crucial role in the accuracy of evaluation results. In this paper, when constructing the evaluation index system, we strictly follow the five basic principles of systematicity, objectivity, measurability, scientificity, and hierarchy. Combining many references and expert opinions, a total of 11 individual indicators are selected which finally form a progressive evaluation index system as shown in Figure 1.

In Figure 1, x_11_ is the index of the insulation resistance test, x_12_ is the index of the pressure-tight test, and x_13_ is the index of the pulse current. x_21_ is the index of the leakage current, x_22_ is the index of the dielectric loss angle, x_23_ is the index of the core temperature of the cables, x_24_ is the index of the partial discharge, x_31_ is the index of the operating life, x_32_ is the index of the operating environment, x_33_ is the index of the load condition, and x_34_ is the index of the history of the fault.

### 4.2. State Space

In this paper, the operating status of the mining cables is classified as severe, abnormal, attention, and normal, denoted as *s_k_*(*k* = 1, 2, 3, 4). Subsequently, the boundary values *c_1_*, *c_2_*, and *c_3_* of adjacent states are determined using the Weibull distribution model based on real-time and historical data to determine the boundary interval (*d*_min_, *d*_max_)k of the kth state level as shown in Table 1. Where *d*_min_ and *d*_max_ are the left and right values of the kth bounding interval, respectively.

In view of the inherent vagueness of the division of state levels and the randomness of the appearance of each state, for this reason, this paper uses a cloud model to portray each state level, i.e., the state cloud. The grade boundary interval and the maximum possible interval of the kth state cloud (*Ex_k_* − 3*En_k_*, *Ex_k_* + 3*En_k_*) form an equation relationship, as shown in Equation (9), from which the expectation (*Ex_k_*) and the entropy (*En_k_*) of each state cloud can be obtained. At the same time, the distribution range of each state cloud is constrained by the limit condition of cloud image fogging, as shown in Formula (10), to obtain the super entropy (*He_k_*) of each state cloud:

(9) $$\{\begin{array}{c} Ex_{k}-3En_{k}=d_{\min}\\ Ex_{k}+3En_{k}=d_{\max} \end{array}\Rightarrow \{\begin{array}{c} Ex_{k}=\frac{d_{\min}+d_{\max}}{2}\\ En_{k}=\frac{d_{\max}-d_{\min}}{6} \end{array}$$

(10) $$He_{k}=\frac{En_{k}}{18}$$

where *Ex_k_* represents the point value that best reflects the *k*-th state level; *En_k_* represents the measured range of the *k*-th state level; *He_k_* represents the degree of cohesion of the data in the *k*-th state.

Send *Ex_k_*, *En_k_*, and *He_k_* into the forward cloud generator to randomly generate the N cloud droplets (*g*, *μ_k_(g*)), which can be visualized in the form of clouds for each operating state. The steps for generating cloud drops in the forward cloud generator are as follows:

Step 1: Enn = Randn(*En_k_, He_k_*). That is, *En_k_* is the expectation and *He_k_* is the standard deviation to generate a normally distributed random number *Enn*.

Step 2: g = Randn(*Exk*, *Enn*). That is, *Exk* is the expectation and *Enn* is the standard deviation to generate a normally distributed random number g.

Step 3: $\mu_{k}(g)=\exp\left[-\frac{\left(g-Ex_{k}\right)}{2{\left(Enn\right)}^{2}}\right]$. The membership is calculated by this equation, and the number pair (*g*, *μ_k_(g*)) represents a cloud droplet distributed over the theoretical domain.

Step 4: Repeat steps 1 to 3 until enough cloud droplets are generated (generally, N is 5000) to restore different operating states in the form of cloud models.

**Figure 2:**
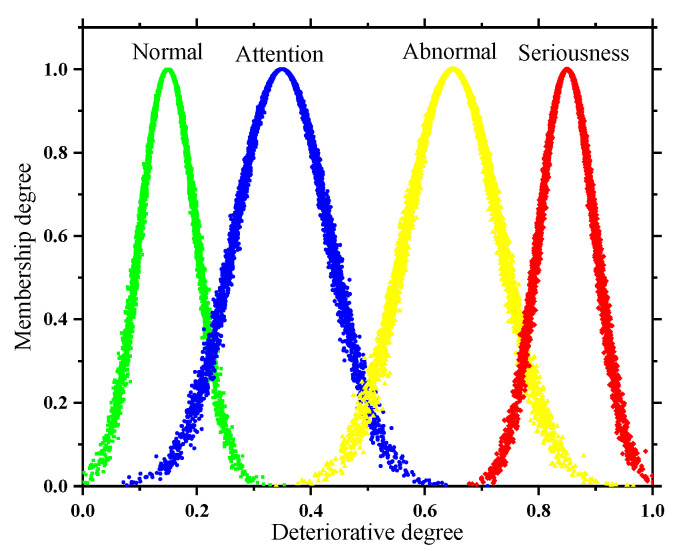
State space of quantitative indicator.

**Figure 3:**
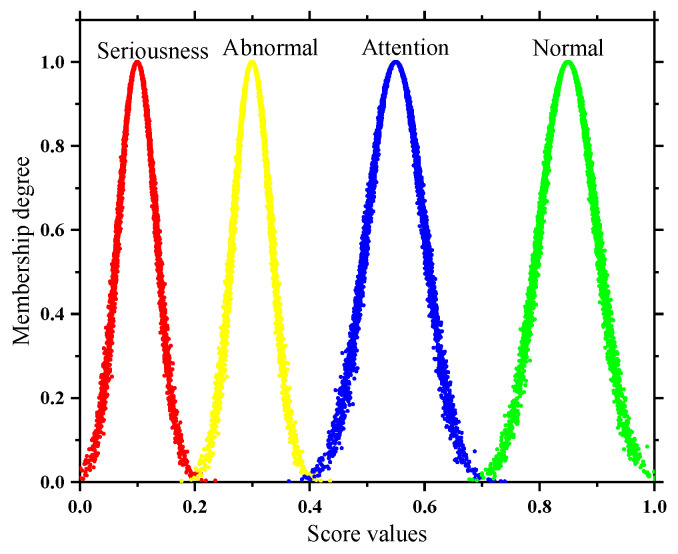
State space of qualitative indicator.

In addition, the subsequent solution of the quantitative and qualitative index membership in this paper is not the same, so two types of state spaces are formed as shown in Figure 2 and Figure 3. In order to take into account the deterioration process of the quantitative index and the tolerance of the equipment to potential adverse factors, the adjacent state clouds in the quantitative space have a certain degree of transition trend; however, the qualitative space is only used as the limit measure, so the adjacent state clouds in the qualitative space are independent of each other.

### 4.3. Quantitative Indicators

**Figure 4:**
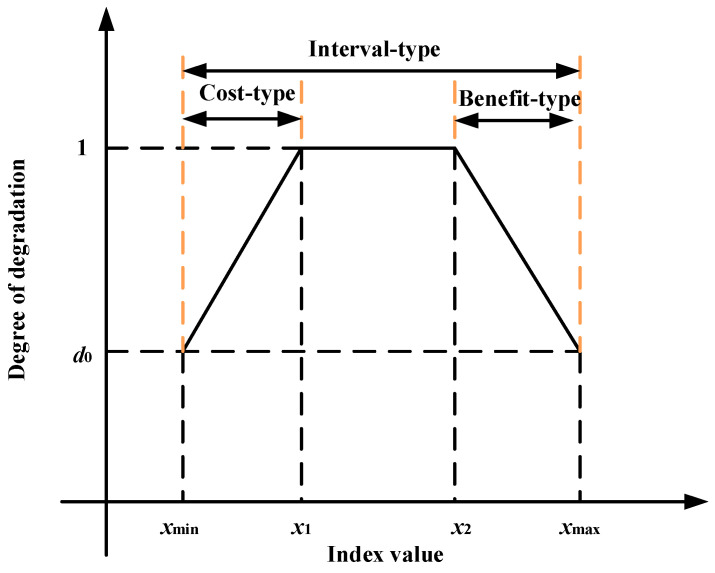
Degradation calculation of different types of indicators.

In this paper, before determining the membership degree of quantitative indicators, the degradation degree function is introduced in order to normalize them and transform them uniformly to the range of [0, 1]. The quantitative indicators within the evaluation system can be divided into three categories: cost type (the smaller the measured value is, the better), benefit type (the larger the measured value is, the better), and interval type (the more centered the measured value is, the better), which are synthetically represented in Figure 4 and Equation (11) in this paper:

(11) $$d=\{\begin{array}{c} \begin{array}{cc} d_{0}+\frac{1-d_{0}}{x_{1}-x_{\min}}(x-x_{\min}) & x_{\min}\leq x\leq x_{1}\\ 1 & x_{1}\leq x\leq x_{2} \end{array}\\ \begin{array}{cc} d_{0}+\frac{d_{0}-1}{x_{\max}-x_{2}}(x-x_{2}) & x_{2}\leq x\leq x_{\max}\\ 0 & x\notin [x_{\min},x_{\max}] \end{array} \end{array}$$

where *x* is the measured value of the quantitative index; *x*_min_ and *x*_max_ are the left and right values of the warning range; *x*_1_ and *x*_2_ are the left and right values of the allowed range; *d* is the degradation degree; and *d*_0_ is the lower limit of the warning range.

After the degradation degree is calculated, the membership degree can be obtained by substituting the result into the expected curve Formula (12) of each state cloud in the quantitative space:

(12) $${r_{k}}^{\left(1\right)}=\exp\left[-\frac{\left(d-Ex_{k}\right)}{2{\left(En_{k}\right)}^{2}}\right]$$

where *r_k_*^(1)^ is the membership of the quantitative index in the *k*-th state.

### 4.4. Qualitative Indicators

**Figure 5:**
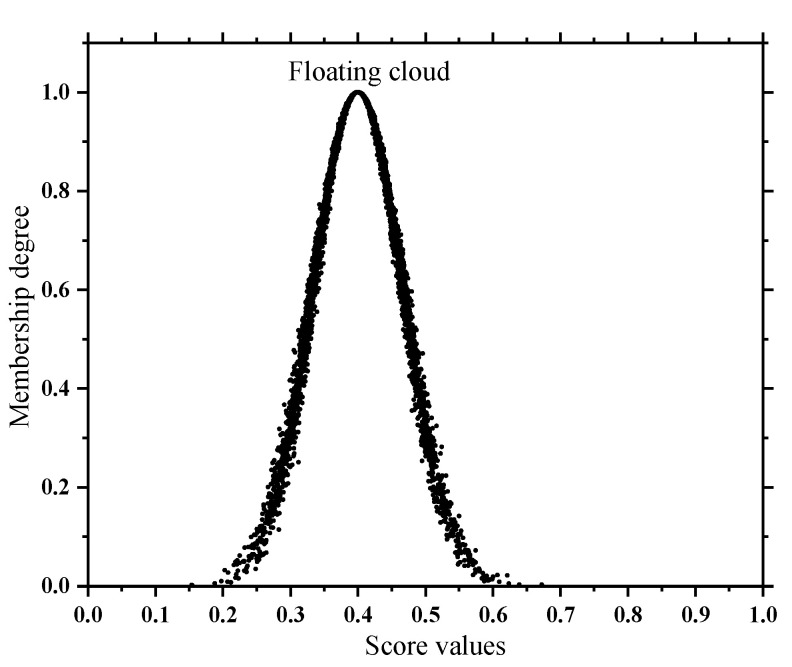
Floating cloud.

Unlike quantitative indexes, the qualitative index membership is further determined only after the experts give their scores empirically by combining the field inspection situation with the test results. In this paper, to weaken the influence of subjectivity in a single expert, h (h = 10 in this paper) experts are invited to score; then numerical feature values are extracted from the discrete scoring results by an inverse cloud generator (Equations (13)–(15)) and then combined with a forward cloud generator to present them visually. The results are called floating clouds, as shown in Figure 5. The more discrete the cloud droplets of the floating cloud are, the greater the degree of disagreement among the experts.

(13) $$Ex_{f}=\frac{1}{h}\sum_{j=1}^{h}p_{j}$$

(14) $$En_{f}=\sqrt{\frac{\pi }{2}}\times \frac{1}{h}\sum_{j=1}^{h}\left|p_{j}-Ex_{f}\right|$$

(15) $$He_{f}=\sqrt{\frac{1}{h}\sum_{j=1}^{h}{\left(p_{j}-Ex_{f}\right)}^{2}-{\left(En_{f}\right)}^{2}}$$

where *p_j_* is the rating given by the *j*-th expert (1-point scale); *Ex_f_*, *En_f_*, and *He_f_* are the expectation, entropy, and hyperentropy of the floating cloud, respectively.

At the same time, in view of the atomization nature of the cloud model (Equation (16)), it is suitable for the consensus judgment of group cognition and can be used as a critical condition in order to judge whether the scoring result of group experts is reasonable; and, if it is not satisfied, it will be re-scored:

(16) $$He_{f}<En_{f}/3$$

**Figure 6:**
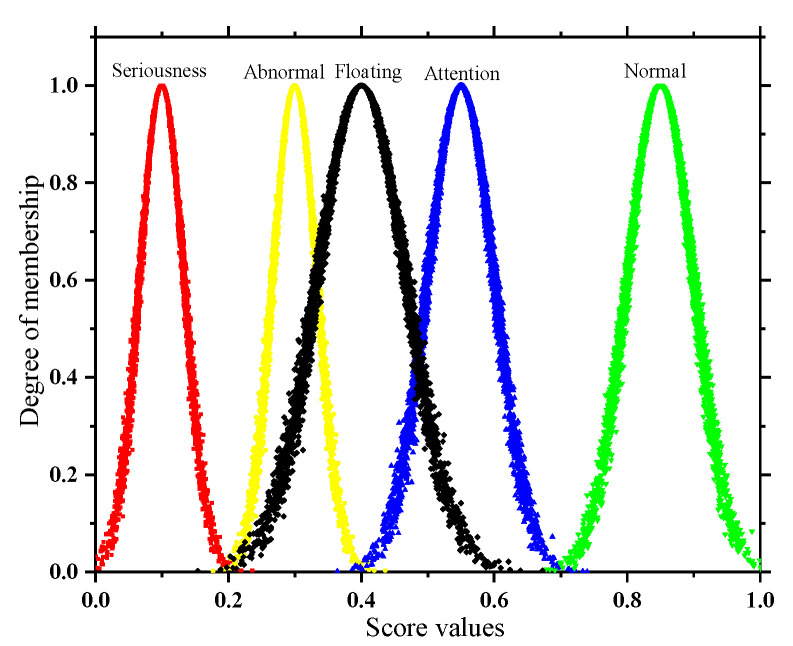
The degree of overlap between the floating cloud and the state cloud.

The greater the degree of overlap between the floating cloud and the *k*-th state cloud in the space (as shown in Figure 6), the stronger the correlation is between the two. Therefore, this paper uses the Formula (17) to calculate the overlapping area O*_k_* of the floating cloud and the *k*-th state cloud. In Figure 6, O_2_ and O_3_ are the overlapping areas of the floating cloud, the abnormal cloud, the floating cloud, and the attention cloud, respectively; the standardized O*_k_* is used as the membership degree of the qualitative index, as shown in Formula (18):

(17) $$O_{k}=\{\begin{array}{cc} \int_{-\infty }^{p_{0}}u_{f}dt+\int_{p_{0}}^{+\infty }u_{k}dt & p_{0}<Ex_{f}\\ \int_{-\infty }^{p_{0}}u_{k}dt+\int_{p_{0}}^{+\infty }u_{f}dt & p_{0}\geq Ex_{f} \end{array}$$

(18) $$r_{k}^{\left(2\right)}=\frac{2O_{k}}{\sqrt{2\pi }(En_{f}+En_{k})}$$

where *r_k_*^(2)^ is the membership of the qualitative indicator in the *k*th state, however, it should be noted that *r_k_*^(1)^ and *r_k_*^(2)^ are only the distinctions between the membership of quantitative and qualitative indicators and all subsequent use of *r_k_* to indicate the membership of an indicator in the *k*-th state level; *u_f_* and *u_k_* are the expectation curves of the *k*-th state cloud in the floating cloud and the qualitative space, respectively; *p*_0_ is the intersection value of the two curves.

### 4.5. Evaluation Algorithm Process

**Figure 7:**
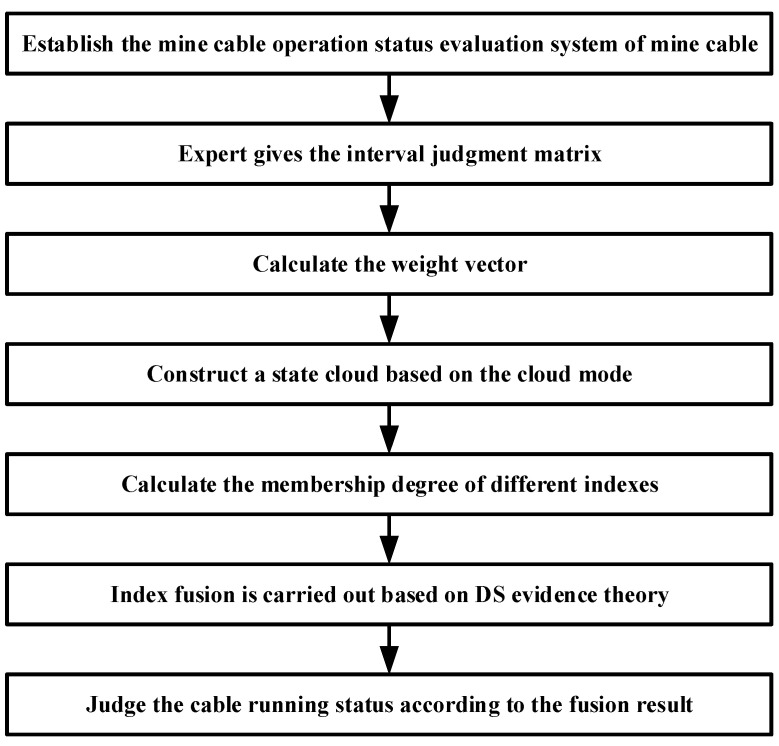
Evaluation algorithm flowchart.

In this paper, based on the principle of AHP algorithm, the evaluation system of mining cable operation status is established based on a combination of expert opinions and literature references, and the weight vectors of indicator layer and criterion layer are calculated according to the improved weight calculation method. According to the respective characteristics of quantitative and qualitative indicators, different methods are adopted to calculate the membership degree of the two types of indicators; finally, the indicator weights are realized according to the D-S evidence theory and membership fusion according to the D-S evidence theory. The specific process is as shown in Figure 7:

Before conducting evidence fusion, it is necessary to standardize the identification framework of the identification object *θ* = {serious state, abnormal state, attention state, normal state, uncertain state} = {*A_t_*}, *t* = 1, 2, 3, 4, 5; then, treat each indicator as evidence and construct the basic confidence assignment function with a generalized fuzzy number for it [26]. Considering that there are differences in the importance degree for each evidence, again, in this paper, we also add the importance factor to its correction, as shown in Equation (19):

(19) $$m_{i}(A_{t})=\{\begin{array}{cc} \beta_{i}\frac{r_{ik}}{\sum_{k=1}^{4}r_{ik}+\left(1-\max\left\{r_{ik}\right\}\right)} & t\in \left[1,4\right]\\ 1-\sum_{t=1}^{4}m_{i}(A_{t}) & t=5 \end{array}$$

(20) $$\beta_{i}=\lambda_{i}\frac{\omega_{i}}{\omega_{\max}}$$

where *r_ik_* is the membership degree of the *i*-th indicator in the *k*-th state; *m_i_*(*A_t_*) is the trust degree of the ith indicator within *θ*; *β_i_* is the importance factor of the i-th evidence; *λ_i_* is the priority trustworthiness coefficient, usually taken as 0.9; and *ω*_max_ is the maximum value of the combination weight.

Finally, according to the synthesis rules of evidence theory (Formulae (21) and (22)), the fusion is carried out step-by-step. In order to solve the problem of the poor status of the base layer indicators that cannot be shown in the final fusion results, this paper proposes the concept of a deviation coefficient *ζa*, as shown in Formula (23):

(21) $$m(B_{t})=\frac{\sum_{\cap A_{t}=B}\prod_{t\leq i\leq 5}m_{i}(A_{t})}{1-K}$$

(22) $$K=\sum_{\cap A_{t}=\emptyset }\prod_{t\leq i\leq 5}m_{i}(A_{t})$$

(23) $$\xi_{a}=\left|K_{Ri}-K_{R}\right|$$

where *K_Ri_* and *K_R_* are the factor layer conflict coefficient and target layer conflict coefficient, respectively, which can be obtained from Equation (22); *ζ_a_* is the deviation coefficient of the *a*-th indicator in the factor layer, and if *ζ_a_* > 0.05, the state of the *a*-th indicator is taken as the final state of the cable, otherwise, the maximum value within the fusion result of the target layer is taken as the final state of the terminal.

## 5. Numerical Work

In order to verify the feasibility of the method proposed in this paper, we used the operational data of a 6 kV cable of a coal mine grid as the basis to determine the operational status of this cable and analyzed the superiority of the method proposed in this paper with the actual calculation results.

### 5.1. Weight Vector Calculation

Due to the limitation of space, the detailed numerical calculation of the weight vector calculation method is not presented in this paper (the detailed numerical calculation has been introduced in the previously published literature [22]). In order to save time in the calculation, we have written an arithmetic program for the weight calculation algorithm using MATLAB (see Supplementary Materials), into which we only need to input the interval judgment matrix reflecting the relative importance among the indicators provided by the experts to obtain the weight vector that we are seeking. The weight vectors of the indicators in each layer are calculated as follows:

$$W=\left[\begin{array}{ccc} 0.1637 & 0.5390 & 0.2973 \end{array}\right]$$

$$W_{1}=\left[\begin{array}{ccc} 0.2493 & 0.1872 & 0.5635 \end{array}\right]$$

$$W_{2}=\left[\begin{array}{cccc} 0.1102 & 0.2801 & 0.0760 & 0.5337 \end{array}\right]$$

$$W_{3}=\left[\begin{array}{cccc} 0.2804 & 0.1249 & 0.5230 & 0.0717 \end{array}\right]$$

*W* is the weight vector of each indicator in the criterion layer, and *W*_1_, *W*_2_, *W*_3_ are the weight vectors of each indicator in the base layer, respectively.

### 5.2. Fuzzy Evalution Matrix

**Figure 8:**
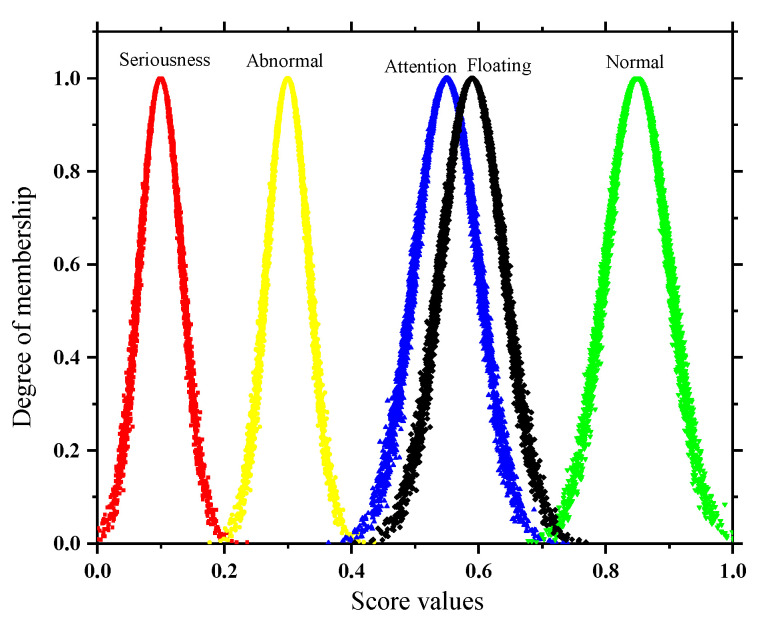
Membership degree of x_31_.

**Figure 9:**
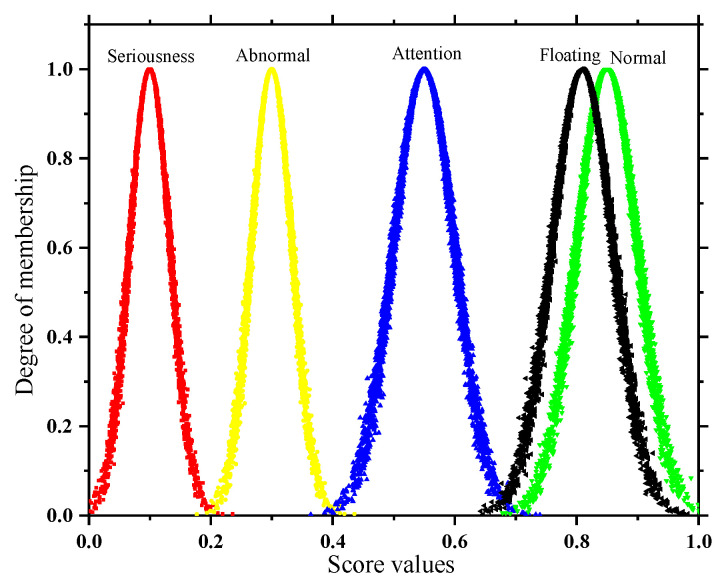
Membership degree of x_32_.

**Figure 10:**
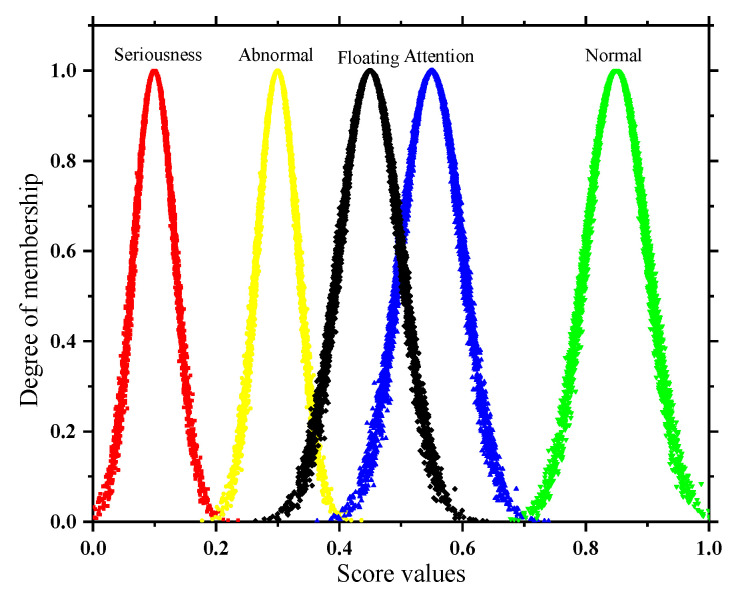
Membership degree of x_33_.

**Figure 11:**
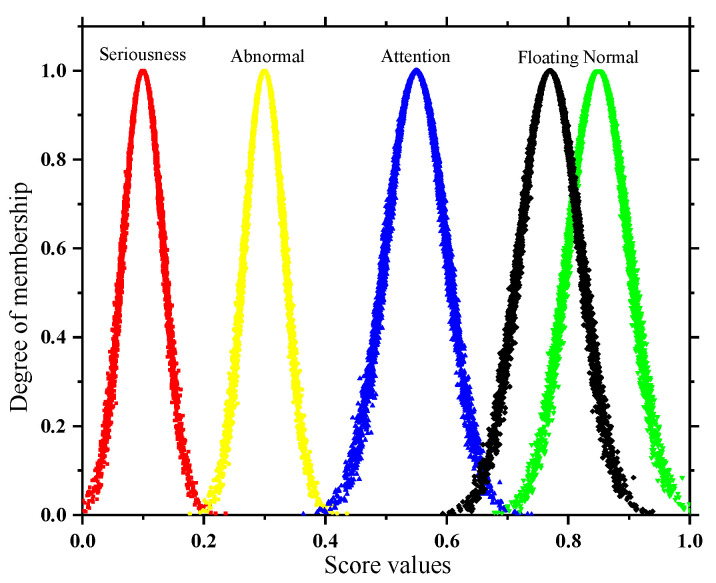
Membership degree of x_34_.

The quantitative and qualitative index membership of the base layer can be calculated according to Formulas (12) and (18), respectively, as shown in Table 3. In this paper, in order to show the feasibility and superiority of the requested qualitative index membership, the results of the membership of x_31_, x_32_, x_33_, and x_34_ under the operating condition information are compared using different methods, as shown in Table 4. Through Table 4, it is easy to find that the most proximate state levels of x_31_, x_32_, x_33_, and x_34_ are identical under the three methods, but the method in this paper is more convenient for us to visually determine the proximate state levels of the four indicators, as shown in Figure 8, Figure 9, Figure 10 and Figure 11. In addition, the fuzzy statistics and the gray theory assign the possibility of x_31_, x_32_, x_33_, and x_34_ to four pre-set state levels completely, i.e., the sum of the membership degree of x_31_, x_32_, x_33_, and x_34_ is homogeneously normalized. Since the existing state levels are not carefully divided, there are deviations between the state space model and the actual model. Experts will express it with an accurate numerical value within the maximum possible range of a certain state and ignore the occurrence of the remaining states. In view of the above considerations, this paper uses the overlap between the floating cloud and the qualitative space in order to reduce the interference of the above factors and make the obtained results more conservative.

### 5.3. Voltage Safety Level Judgement

According to the relevant principles of D-S evidence theory, the relevant parameters of the factor layer indicators can be calculated by combining Equations (19) and (20), as shown in Table 5. Combined with Table 5, the target layer *m*(*B*) = {0, 0.0059, 0.1261, 0.6805, 0.1875} can be calculated by Formula (21). According to the principle of the maximum trust degree, it can be determined that the operating state of the cable is in a normal state and there is also a weak degree of trust in the attention state and abnormal state. Therefore, it is necessary to continue to analyze the conflict coefficient between the factor layer and the target layer. Taking the conflict coefficient of the target layer as the reference value and by comparing the conflict coefficients of x_1_, x_2_, and x_3_ with the reference value, the deviation coefficient of each index of the factor layer can be calculated by Formula (23), and it is found that the deviation coefficients are all less than 0.05, so there is no need to correct the judgment result.

## 6. Conclusions

The safe and stable operation of mining cables directly affects the safe production of coal mines. If we can accurately assess the operating status of mining cables, we can prevent problems before they occur and respond in time before accidents happen. In order to realize the accurate evaluation of the operating state of the mining cable, this paper proposes a comprehensive evaluation method based on AHP, membership cloud theory, and D-S evidence theory. Considering the ambiguity and randomness of the state level, this paper introduces the membership cloud theory to visualize the cable running state in the form of a cloud. When calculating the membership degree of the qualitative index, the overlapping degree of the floating cloud and the state space is used as the membership degree of the qualitative index which can more intuitively reflect the relationship between the expert score and the state level. In the fusion of index weight and membership degree, this paper replaces the traditional fuzzy synthesis algorithm with D-S evidence theory. The main conclusions are as follows:

(1) In this paper, by introducing the fogging condition of the cloud model, we objectively verify the rationality of the subjective scoring of experts and then realize the intuitive comparison between the actual distribution of qualitative indicators (i.e., floating cloud) and the standard distribution (i.e., qualitative space).
(2) Compared with fuzzy statistics and gray theory, the qualitative index membership degree calculation method proposed in this paper can make the membership degree calculation result more conservative and intuitive.
(3) The D-S evidence theory can effectively integrate the index weight and membership degree and, at the same time, avoid a situation where the abnormal state of the underlying index is covered by the deviation coefficient of the conflict coefficient, thereby improving the correctness of the judgment result.

## Figures and Tables

**Table 1:** Meaning of the scale.

Scale	Meaning
1	The two indicators are equally important
3	The former indicator is slightly more important than the latter
5	The former indicator is more important than the latter
7	The former indicator is certainly more important than the latter
9	The former indicator is much more important than the latter
2, 4, 6, 8	The judgment is between the two adjacent judgments

**Table 2:** Random consistency index.

*n*	1	2	3	4	5	6	7	8
RI	0	0	0.58	0.90	1.12	1.24	1.32	1.45

**Table 3:** Index membership of base layer.

Index	Seriousness	Abnormal	Attention	Normal
x_11_	0	0	0.0736	0.7228
x_12_	0	0	0.0491	0.8237
x_13_	0	0	0.0564	0.7683
x_21_	0	0	0.0518	0.7746
x_22_	0	0	0.0795	0.6938
x_23_	0	0	0.0432	0.8523
x_24_	0	0	0.0503	0.7826
x_31_	0	0	0.6979	0.0798
x_32_	0	0	0.0834	0.6779
x_33_	0	0.1053	0.4573	0
x_33_	0	0	0.0969	0.5768

**Table 4:** Membership solution results of x_31_, x_32_, x_33_, and x_34_ under different methods.

Method	The Method of This Paper				Fuzzy Statistics				Grey Theory			
Status	s_1_	s_2_	s_3_	s_4_	s_1_	s_2_	s_3_	s_4_	s_1_	s_2_	s_3_	s_4_
x_31_	0	0	0.6979	0.0798	0	0	1	0	0	0	0.8362	0.1638
x_32_	0	0	0.0834	0.6779	0	0	0	1	0	0	0.2084	0.7916
x_33_	0	0.1053	0.4573	0	0	0.2875	0.7125	0	0	0.3173	0.6827	0
x_33_	0	0	0.0969	0.5768	0	0	0	1	0	0	0.7145	0.2885

**Table 5:** Factor layer related parameters.

Index	x_1_	x_2_	x_3_
Weight	0.1637	0.5390	0.2973
Seriousness *m*(*B*)	0	0	0
Abnormal *m*(*B*)	0	0	0.0520
Attention *m*(*B*)	0.0412	0.0788	0.5320
Normal *m*(*B*)	0.7618	0.8212	0.0978
Uncertainty	0.1970	0.1000	0.3182

## Data Availability

Not applicable.

## Supplementary Materials

The following supporting information can be downloaded at: https://www.mdpi.com/article/10.3390/s22197174/s1, Weight Calculation Program of AHP.
