# Supplementary material for: A Comprehensive Operation Status Evaluation Method for Mining XLPE Cables
Source: Sensors (Basel). 2022 Sep 21;22(19):7174. doi: 10.3390/s22197174 (PMC9571771; doi:10.3390/s22197174)
Supplement: Supplementary file 1 [file sensors-22-07174-s001.zip › sensors-1916536-supplementary.pdf]

Supplementary Materials

```
clc;
clear;
A = {1,[2,3],[1/3,1/2],[4,5];[1/3,1/2],1,[1/5,1/4],[2,3];[2,3],[4,5],1,[5,6];[1/5,1/4],[1/3,1/2],[1/6,1/5],1};
Q = 100;
n = 4;
RI = 0.9;
CR_r = 0.1;
p = 0;
for loop = 1:Q
    A_tem = zeros(4,4);
    for i = 1:4
        for j = 1:4
            if i == j
                A_tem(i,j) = 1;
            else
                range = A{i,j};
                a = range(1);
                b = range(2);
                A_tem(i,j) = a + (b-a)*rand;
            end
        end
    end
    [x_tem,y_tem] = eig(A_tem);
    lammda_max = max(diag(y_tem));
    CR_tem = (lammda_max - n)/(RI*(n-1));
    if CR_tem < CR_r
        p = p + 1;
    end
end
in = 0;
gamma = p/Q;
gamma_r = 0.6;
if gamma <= gamma_r
    disp('Need to adjust the element')
else
    flag_loop = true;
    while flag_loop
        R = 100;
        A_all = [];
        CR = [];
        for loop = 1:R
            A_tem = zeros(4,4);
            for i = 1:4
                for j = 1:4
                    if i == j
                        A_tem(i,j) = 1;
                    else
                        range = A{i,j};
                        a = range(1);
                        b = range(2);
                        A_tem(i,j) = a + (b-a)*rand;
                    end
                end
```

```
        end
        A_all = [A_all;A_tem];
        [x_tem,y_tem] = eig(A_tem);
        lammda_max = max(diag(y_tem));
        CR_tem = lammda_max - n*RI/(n-1);
        CR = [CR,CR_tem];
end
in = in + 1;
if in > 2000
        break
end
omega = 10;
CR_copy = CR;
A_omega = {1,0,0,0;0,1,0,0;0,0,1,0;0,0,0,1};
for loop = 1:omega
        [value,index] = min(CR_copy);
        init_row_index = 4*(index-1)+1;
        A_tem = A_all(init_row_index:init_row_index+3,:);
        for i = 1:4
                for j = 1:4
                        if i ~= j
                                if loop == 1
                                        A_omega{i,j} = A_tem(i,j);
                                else
                                        A_omega{i,j} = [A_omega{i,j},A_tem(i,j)];
                                end
                        end
                end
        end
      CR_copy(index) = max(CR_copy)+1;
end
A_new = {1,0,0,0;0,1,0,0;0,0,1,0;0,0,0,1};
for i = 1:4
        for j = 1:4
                if i < j
                        range_tem = A_omega{i,j};
                        a = min(range_tem);
                        b = max(range_tem);
                        A_new{i,j} = [a,b];
                elseif i > j
                        range_tem = A_omega{j,i};
                        a = min(range_tem);
                        b = max(range_tem);
                        A_new{i,j} = [1/b,1/a];
                end
        end
end
width_new = 0;
width_origin = 0;
for i = 1:4
        for j = 1:4
                if i ~= j
                        range_tem = A_new{i,j};
                        width_new_tem = abs(range_tem(2) - range_tem(1));
                        width_new = width_new + width_new_tem;
```

```
                range_tem = A{i,j};
                width_origin_tem = abs(range_tem(2) - range_tem(1));
                width_origin = width_origin + width_origin_tem;
            end
        end
    end
    if width_new <= width_origin*0.1
        flag_loop = false;
    end
end
M0 = eye(4);
for i = 1:4
    for j = 1:4
        if i < j
            range_tem = A_new{i,j};
            M0(i,j) = (range_tem(1) + range_tem(2))/2;
        elseif i > j
            range_tem = A_new{j,i};
            M0(i,j) = 2/(range_tem(1) + range_tem(2));
        end
    end
end
[x_tem,y_tem] = eig(A_tem);
lammda_max = max(diag(y_tem));
CR0 = lammda_max - n*RI/(n-1);
c = 2;
tao = 1;
v_tem = zeros(4,4);
M0_tem = M0;
Mi_tem = zeros(4,4);
ksi_tem = zeros(4,4);
gap = [];
for i = 1:4
    for j = 1:4
        if i ~= j
            range = A_new{i,j};
            gap_tem = range(2) - range(1);
            gap = [gap,gap_tem];
        end
    end
end
vmax = min(gap);
for k = 1:1000
    if k == 1
        for i = 1:4
            for j = 1:4
                if i == j
                    Mi_tem(i,j) = 1;
                else
                    range = A_new{i,j};
                    a = range(1);
                    b = range(2);
                    Mi_tem(i,j) = a + (b-a)*rand;
                end
            end
```

```
            end
            ksi_tem = Mi_tem;
        else
            ksi_tem = ksi_tem + v_tem;
            for i = 1:4
                for j = 1:4
                    if i ~= j
                        range = A_new{i,j};
                        if ksi_tem(i,j) < range(1)
                            ksi_tem(i,j) = range(1);
                        end
                        if ksi_tem(i,j) > range(2)
                            ksi_tem(i,j) = range(2);
                        end
                    else
                        ksi_tem(i,j) = 1;
                    end
                end
            end
            Mi_tem = ksi_tem;
        end
        for i = 1:4
            for j = 1:4
                if i ~= j
                    lammda = rand;
                    value_tem = tao*v_tem(i,j) + c*lammda*(M0_tem(i,j)-Mi_tem(i,j));
                    if value_tem > vmax
                        value_tem = vmax;
                    end
                    if value_tem < -vmax
                        value_tem = -vmax;
                    end
                    v_tem(i,j) = value_tem;
                end
            end
        end
        [x_tem,y_tem] = eig(M0_tem);
        lammda_max = max(diag(y_tem));
        CR_M0 = lammda_max - n*RI/(n-1);
        [x_tem,y_tem] = eig(Mi_tem);
        lammda_max = max(diag(y_tem));
        CR_Mi = lammda_max - n*RI/(n-1);
        if CR_Mi < CR_M0
            M0_tem = Mi_tem;
        end
    end
    matrix_norm = zeros(4,4);
    for j = 1:4
        sum_row_values = sum(M0_tem(:,j));
        for i = 1:4
            matrix_norm(i,j) = M0_tem(i,j)/sum_row_values;
        end
    end
    w_hat = zeros(4,1);
    for i = 1:4
```

```
            w_hat(i,1) = sum(matrix_norm(i,:));
        end
        w = zeros(4,1);
        for i = 1:4
            w(i,1) = w_hat(i,1)/sum(w_hat);
        end
        disp(M0)
        disp(' w: ')
        disp(w)
end
```
